# Eight Weeks of Aerobic Exercise Training Improves Fitness, Metabolic Health, Inflammation, and Intestinal Barrier Integrity in Overweight and Obese Women of Different Age Groups

**DOI:** 10.3390/life15111752

**Published:** 2025-11-14

**Authors:** Tae-Hyung Lee, Hyung-Il Lee, Hee-Tae Roh, Su-Youn Cho

**Affiliations:** 1 Exercise Physiology Laboratory, Department of Physical Education, Yonsei University, Seoul 03722, Republic of Korea; 2 Division of Sports Science, College of Arts and Sports, Sun Moon University, Asan-si 31460, Republic of Korea

**Keywords:** aerobic exercise, obesity, age-related effects, organokines, inflammatory responses, gut integrity

## Abstract

This study investigated the effects of eight weeks of aerobic exercise training on body composition, lipid profiles, organokines (leptin, irisin), inflammatory biomarkers (high-sensitivity C-reactive protein [hs-CRP], interleukin-6 [IL-6]), and intestinal barrier permeability markers (zonulin, lipopolysaccharide-binding protein [LBP]) in overweight and obese women of different age groups. We hypothesized that aerobic exercise would improve cardiorespiratory fitness, body composition, lipid metabolism, and reduce pro-inflammatory responses and intestinal permeability, and that these effects would differ between age groups. A total of 32 participants with a body mass index (BMI) ≥ 23 kg/m^2^ were randomly assigned to one of four groups (*n* = 8 per group): young exercise (YE), young control (YC), middle-aged exercise (ME), and middle-aged control (MC). The intervention consisted of treadmill running for 50 min per session, four times per week, at an intensity corresponding to 65% of the target heart rate (THR), calculated using the Karvonen formula, for a duration of eight weeks. Body composition variables included body weight, BMI, body fat mass (BFM), percentage body fat (PBF), lean body mass (LBM), and maximal oxygen uptake (VO_2_max). Blood samples were analyzed for lipid profiles (total cholesterol [TC], triglycerides [TG], low-density lipoprotein cholesterol [LDL-C], high-density lipoprotein cholesterol [HDL-C]), organokines, inflammatory markers, and intestinal barrier integrity biomarkers. After the intervention, the YE and ME groups exhibited significant reductions (*p* < 0.05) in body weight, BMI, BFM, PBF, TC, TG, LDL-C, leptin, hs-CRP, IL-6, zonulin, and LBP. In contrast, LBM and VO_2_max significantly increased (*p* < 0.05) in both exercise groups. No significant changes were observed in irisin concentrations or HDL-C levels (*p* > 0.05). These results suggest that aerobic exercise training, irrespective of age, is effective in improving cardiorespiratory fitness, body composition, and lipid metabolism, while simultaneously reducing systemic inflammation and is associated with favorable changes in circulating biomarkers of intestinal barrier function in overweight and obese women.

## 1. Introduction

Obesity has markedly increased worldwide and is now regarded as a global pandemic [1,2]. It is associated not only with cardiovascular and metabolic disorders but also with adverse mental health outcomes, including depression, anxiety, and impaired quality of life [2]. It arises from excessive fat accumulation in the body due to an imbalance between energy intake and expenditure [3,4]. The contributing factors include sedentary behavior; insufficient physical activity; high-fat and carbohydrate diets; and genetic, environmental, and age-related influences [5]. Excessive visceral adipose tissue promotes the release of pro-inflammatory cytokines and chemokines, thereby inducing systemic inflammation and oxidative stress and contributing to the onset of metabolic dysfunction, gastrointestinal disorders, and other complications [5,6]. The gastrointestinal tract, which contains approximately 70% of the body’s lymphocytes, is the largest immune organ that plays a central role in immune homeostasis [7]. Consequently, the disruption of the intestinal barrier can lead to systemic inflammation [1].

Systemic inflammation is a hallmark of excessive fat accumulation [8]. Numerous studies have identified obesity as a common pathological feature underlying obesity-related comorbidities, highlighting the potential link between obesity, systemic inflammation, and intestinal barrier dysfunction [1]. Mechanistically, obesity-associated inflammation is thought to result from an imbalance between pro- and anti-inflammatory mediators [9], often driven by the dysregulated secretion of organokines [10]. Organokines are bioactive proteins secreted by adipose tissue, skeletal muscle, and the liver [5,10,11]. Among these, leptin is one of the most extensively studied adipokines with a pro-inflammatory role, whereas irisin, a myokine secreted in response to physical activity, has recently attracted attention for its role in exercise-induced metabolic adaptations and suppression of inflammatory responses [12,13]. Collectively, adipokines and myokines exert beneficial and detrimental effects on metabolic regulation [9,10,14]. Importantly, exercise has been shown to induce favorable changes in both [15]. In addition to stimulating myokine secretion, exercise reduces the incidence and prevalence of chronic diseases [16], with beneficial outcomes reported across resistance, aerobic, and high-intensity training modalities [17]. Aerobic exercise is widely recognized as an effective intervention for obesity that improves body composition, regulates lipid metabolism, and alleviates inflammation [18,19].

Recent evidence suggests that exercise influences gut metabolism positively by reducing systemic inflammation and endotoxemia of intestinal origin [20]. Biomarkers of intestinal barrier function are generally classified as fecal or plasma markers [21]. Elevated plasma levels of zonulin and lipopolysaccharide-binding protein (LBP) are hallmarks of intestinal barrier dysfunction and are strongly associated with obesity-related inflammation and metabolic abnormalities [1,6,21]. Zonulin regulates tight junctions between enterocytes in the small intestine, thereby increasing intestinal permeability [22], whereas LBP binds to cluster of differentiation 14 (CD14) to form CD14–LPS complexes, which activate pro-inflammatory signaling cascades [23]. Elevated LBP has been linked to obesity, type 2 diabetes, and metabolic syndrome [24].

Nevertheless, the effects of aerobic exercise on the intestinal barrier function and its broad role in obesity-related chronic inflammation and metabolic dysfunction remain inconclusive [25,26], with outcomes potentially influenced by age and individual characteristics. In women, physiological changes associated with the pre- and post-menopausal transition—including alterations in body composition, systemic inflammation, mitochondrial function, and intestinal barrier integrity—may substantially affect organokine responses and barrier-related biomarkers [27,28,29]. Furthermore, physiological responses to exercise have been shown to differ across age groups [30,31,32]. These differences are particularly pronounced in women, given the influence of hormonal fluctuations, body composition, and mitochondrial function on menopausal status. However, the role of age-specific variations in shaping exercise-induced changes in organokine levels and intestinal permeability remains unclear.

Therefore, the present study aimed to examine the effects of an 8-week aerobic exercise training on body composition, lipid profiles, organokines, inflammatory markers, and intestinal permeability markers in overweight and obese women stratified by age. These findings are expected to provide insights for the development of tailored exercise interventions to promote women’s health across different stages of life.

We hypothesized that 8 weeks of aerobic exercise would improve body composition, lipid profiles, organokines, inflammatory markers, and intestinal barrier function, and that these effects may differ according to age group.

## 2. Methods

### 2.1. Participants

This prospective intervention trial recruited 32 overweight and obese women (BMI ≥ 23 kg/m^2^), including 16 young premenopausal adults (aged 20–33 years) and 16 middle-aged postmenopausal adults (aged 50–64 years) who voluntarily agreed to participate. Participants were recruited as volunteers through community advertisements in S city, Korea, and bulletin boards at Y University. All participants were healthy, non-smoking, free from cardiovascular or musculoskeletal disorders that could limit exercise participation, and were physically capable of completing moderate-intensity aerobic exercise. None of the participants was taking medications or dietary supplements known to affect metabolism or inflammation. No specific dietary control or monitoring was implemented during the intervention period. Sample size estimation was performed using G*Power software (version 3.1.9.7; Heinrich-Heine-University, Düsseldorf, Germany) with the following parameters: effect size (ES) = 0.40, α = 0.05, and statistical power (1−β) = 0.95. The chosen effect size (0.40) was based on Cohen’s conventional definition of a medium-to-large effect size [33]. The analysis indicated that at least eight participants per group were required. Accordingly, the participants were randomly assigned to one of four groups stratified by age: young exercise (YE), young control (YC), middle-aged exercise (ME), and middle-aged control (MC). Randomization was performed using a computer-generated random sequence, and allocation was concealed in sealed opaque envelopes to ensure transparency.

The eligibility criteria were as follows: women with a BMI ≥ 23 kg/m^2^, free from physical limitations that could restrict exercise participation, and without cardiovascular or musculoskeletal disorders. The exclusion criteria were a diagnosis of metabolic diseases other than overweight or obesity, participation in regular exercise programs, current use of medications or supplements that could influence study outcomes, and smoking.

All participants were fully informed of the study objectives, procedures, and potential benefits and risks, and they all provided written informed consent prior to enrollment. The study protocol was approved by the Institutional Review Board of Yonsei University (IRB no. 1040917-201506-BR-158-04) and was conducted in accordance with the principles outlined in the most recent revision of the Declaration of Helsinki. The baseline physical characteristics of the participants are presented in Table 1. All participants met the inclusion criteria and successfully completed the 8-week intervention without dropout. No missing data occurred. To minimize potential confounding effects, participants were instructed to maintain their usual diet and daily activities throughout the study.

### 2.2. Anthropometric Measurements and Maximal Oxygen Uptake

Height and body weight were measured using a stadiometer equipped with an integrated scale (BSM330; Biospace, Seoul, Republic of Korea). Body composition variables, including body fat mass (BFM), percentage body fat (PBF), and lean body mass (LBM), were assessed using bioelectrical impedance analysis (InBody 720; Biospace, Seoul, Republic of Korea).

Maximal oxygen uptake (VO_2_max) was measured on a motorized treadmill (TM65; Quinton, Bothell, WA, USA) with a metabolic measurement system (TrueOne 2400; ParvoMedics, Sandy, UT, USA) following the Modified Bruce Protocol [34]. The test was conducted under close supervision and was terminated at the point of volitional exhaustion, following a symptom-limited maximum effort criterion.

### 2.3. Exercise Training Intervention

The aerobic exercise program was performed four times per week for 8 weeks, with each session lasting 60 min. Each session comprised 50 min of treadmill running, preceded by a 5 min warm-up and followed by a 5 min cool-down consisting of stretching and light walking.

Exercise intensity during treadmill running was set at 65% of the target heart rate (THR), calculated using the Karvonen formula, and was maintained at the same level throughout the 8 weeks. No progressive overload was applied to ensure consistency of the intervention. The intervention was conducted at the fitness center of Y University, and all sessions were supervised by trained research staff. Heart rate was continuously monitored using a heart rate monitor (FT2; Polar Electro, Kempele, Finland). All participants attended every scheduled session, resulting in an adherence rate of 100%.

The 8-week aerobic training protocol (4 sessions per week, 60 min per session, at 65% THR) was selected based on the American College of Sports Medicine (ACSM) guidelines for improving cardiorespiratory fitness and metabolic health. In addition, previous studies in overweight and obese populations have demonstrated that similar exercise prescriptions are effective in reducing body fat, improving lipid profiles, and lowering inflammatory markers [35,36]. Prior to the intervention, all participants completed familiarization sessions to ensure they could safely perform 50 min of treadmill running at the prescribed intensity.

To minimize potential confounding factors, participants were instructed to maintain their habitual dietary habits throughout the 8-week intervention. They were also asked to refrain from alcohol consumption and to avoid any additional structured exercise beyond the assigned program. The control groups, young control (YC) and middle-aged control (MC), were not permitted to participate in any organized exercise training during the study period.

### 2.4. Blood Sampling and Biochemical Analysis

Venous blood samples were collected twice, at baseline and post-intervention, from the antecubital vein using a 22-gauge needle, a serum separator tube (SST), and an ethylenediaminetetraacetic acid (EDTA) tube, with 10 mL drawn for each collection. Post-intervention blood samples were collected 48 h after the last exercise session to minimize the influence of acute exercise effects. All samples were obtained in a resting state after a minimum of 12 h of overnight fasting. Following collection, blood was centrifuged at 3000 rpm for 15 min at 4 °C to separate serum and plasma. Aliquots were stored at −80 °C until analysis of lipid profiles (total cholesterol [TC], triglycerides [TG], low-density lipoprotein cholesterol [LDL-C], and high-density lipoprotein cholesterol [HDL-C]), organokines (leptin, irisin), inflammatory biomarkers (high-sensitivity C-reactive protein [hs-CRP], interleukin-6 [IL-6]), and intestinal barrier integrity biomarkers (zonulin, lipopolysaccharide-binding protein [LBP]).

Serum lipid profiles (TC, TG, LDL-C, and HDL-C) were measured using enzymatic colorimetric assay kits; Triglycerides Gen. 2 (No. 11877771 216), Cholesterol Gen. 2 (No. 11875500 216), LDL-cholesterol Plus 2nd Generation (No. 03039773 190), and HDL-cholesterol Plus 3rd Generation (No. 06393794 190) were obtained from Roche Diagnostics (Mannheim, Germany). Analyses were conducted using the Modular Analytics System (PE; Roche Diagnostics, Mannheim, Germany), in accordance with the manufacturer’s instructions.

Among organokines, we focused on leptin and irisin because they are among the most widely studied with strong relevance to obesity, exercise responses, and metabolic health, and their assays are feasible and reliable for clinical research. Plasma irisin concentrations were determined using an enzyme-linked immunosorbent assay (ELISA; Irisin Recombinant [Human, Mouse, Rat] EIA Kit, No. EK-067-29; Phoenix Pharmaceuticals, Burlingame, CA, USA). Serum leptin levels were measured by means of a radioimmunoassay (RIA; Human Leptin RIA Kit, No. HL-81K; Millipore, Billerica, MA, USA) and quantified using a γ-counter (COBRA 5010 Quantum; Packard Instrument Co., Downers Grove, IL, USA).

Plasma hs-CRP, IL-6, zonulin, and LBP levels were measured using ELISA kits: Human CRP Quantikine ELISA Kit (No. DCRP00; R&D Systems, Minneapolis, MN, USA); a Human IL-6 Quantikine ELISA Kit (No. D6050; R&D Systems); a Human Zonulin ELISA Kit (No. CSB-EQ027649HU; CUSABIO, Wuhan, China); and Human LBP DuoSet ELISA Kit (No. DY870-05; R&D Systems). All assays were performed according to the manufacturers’ instructions.

According to the manufacturers’ information, the intra- and inter-assay coefficients of variation (CVs) for all assays were <10%, indicating acceptable assay reliability.

### 2.5. Statistical Analyses

All statistical analyses were performed using SPSS software (version 26.0; IBM Corp., Armonk, NY, USA). Data are presented as mean ± standard deviation. To evaluate the effects of the intervention, a two-way repeated-measures analysis of variance (ANOVA) was conducted with group (YE, YC, ME, and MC) as the between-subject factor and time (baseline vs. post-intervention) as the within-subject factor. When a significant interaction was observed, one-way ANOVA was applied to compare differences between groups at each time point, and paired *t*-tests were used to assess within-group changes over time. Post hoc pairwise comparisons were conducted where appropriate. Effect sizes for ANOVA were calculated as partial η^2^. No missing data occurred. Statistical significance was set at *p* < 0.05. The reporting of this randomized controlled trial followed the Consolidated Standards of Reporting Trials (CONSORT) guidelines [37]. In addition, to enhance clarity and transparency, the reporting also considers key items from the STROBE checklist for cross-sectional studies.

## 3. Results

### 3.1. Changes in Body Composition and Maximal Oxygen Uptake

**Figure 1:**
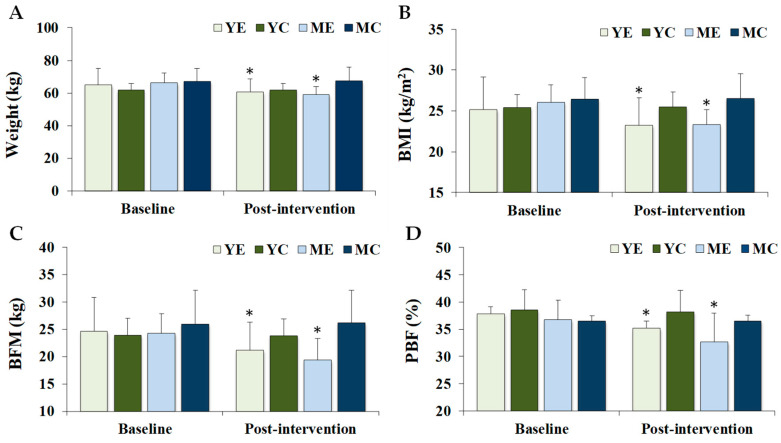

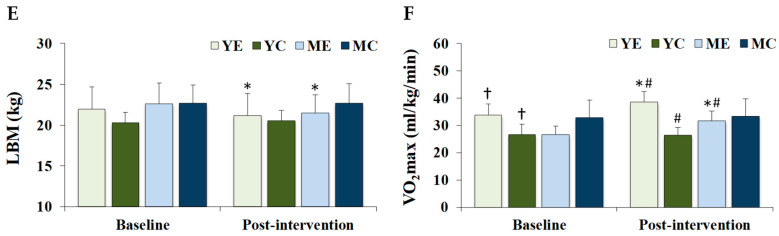
Changes in the body composition and maximal oxygen uptake. Values are presented as mean ± standard deviation. (**A**) weight; (**B**) BMI, body mass index; (**C**) BFM, body fat mass; (**D**) PBF, percentage body fat; (**E**) LBM, lean body mass; (**F**) VO_2_max, maximal oxygen uptake; YE, young exercise; YC, young control; ME, middle-aged exercise; MC, middle-aged control; * *p* < 0.05 vs. baseline within the same group; ^†^ *p* < 0.05 vs. ME and MC groups; ^#^ *p* < 0.05 vs. MC group.

Figure 1 shows the changes in the body composition and maximal oxygen uptake. Significant time × group interactions were observed for weight (F (3, 28) = 13.500, *p* < 0.001, partial η^2^ = 0.591), BMI (F (3, 28) = 24.593, *p* < 0.001, partial η^2^ = 0.599), BFM (F (3, 28) = 9.876, *p* < 0.001, partial η^2^ = 0.514), PBF (F (3, 28) = 8.304, *p* < 0.001, partial η^2^ = 0.471), LBM (F (3, 28) = 76.494, *p* < 0.001), and VO_2_max (F (3, 28) = 93.272, *p* = 0.001, partial η^2^ = 0.425). Post hoc analyses indicated that body weight, BMI, BFM, and PBF significantly decreased in the YE and ME groups compared to baseline (*p* < 0.05), whereas LBM and VO_2_max significantly increased in the same groups (*p* < 0.05). At baseline, VO_2_max was significantly higher in the YE and YC groups than in the ME and MC groups (*p* < 0.05). At post-intervention, VO_2_max values in the YE, YC, and ME groups were significantly higher than those in the MC group (*p* < 0.05).

### 3.2. Changes in Lipid Profile

**Figure 2:**
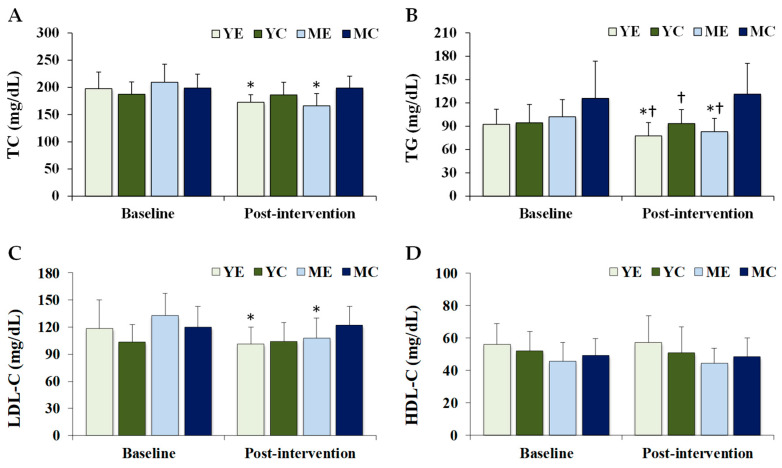
Changes in the serum lipid profiles. Values are presented as mean ± standard deviation. (**A**) TC, total cholesterol; (**B**) TG, triglycerides; (**C**) LDL-C, low-density lipoprotein cholesterol; (**D**) HDL-C, high-density lipoprotein cholesterol; YE, young exercise; YC, young control; ME, middle-aged exercise; MC, middle-aged control; * *p* < 0.05 vs. baseline within the same group; ^†^ *p* < 0.05 vs. MC group.

Figure 2 shows the changes in the serum lipid profiles. Significant time × group interactions were observed for TC (F (3, 28) = 6.956, *p* = 0.001, partial η^2^ = 0.427), TG (F (3, 28) = 3.982, *p* = 0.018, partial η^2^ = 0.321), and LDL-C (F (3, 28) = 5.537, *p* = 0.004, partial η^2^ = 0.372), whereas HDL-C showed no significant effect (F (3, 28) = 0.299, *p* = 0.826, partial η^2^ = 0.031). Post hoc analyses demonstrated that TC, TG, and LDL-C levels decreased significantly in the YE and ME groups compared to baseline (*p* < 0.05). Post-intervention TG levels were significantly lower in the YE, YC, and ME groups than in the MC group (*p* < 0.05).

### 3.3. Changes in Organokines

**Figure 3:**
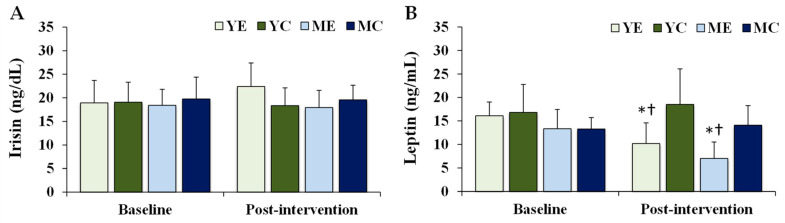
Changes in the plasma organokines. Values are presented as mean ± standard deviation. (**A**) irisin; (**B**) leptin; YE, young exercise; YC, young control; ME, middle-aged exercise; MC, middle-aged control; * *p* < 0.05 vs. baseline within the same group; ^†^ *p* < 0.05 vs. YC group.

Figure 3 shows the changes in the plasma organokines. Significant time × group interaction was observed for leptin (F (3, 28) = 7.167, *p* = 0.001, partial η^2^ = 0.534), whereas no significant effect was observed for irisin (F (3, 28) = 1.368, *p* = 0.273, partial η^2^ = 0.128). Post hoc analyses showed that leptin levels were significantly decreased in the YE and ME groups compared to baseline (*p* < 0.05). Post-intervention leptin levels in the YE and ME groups were significantly lower than those in the YC group (*p* < 0.05).

### 3.4. Changes in Inflammatory Biomarkers

**Figure 4:**
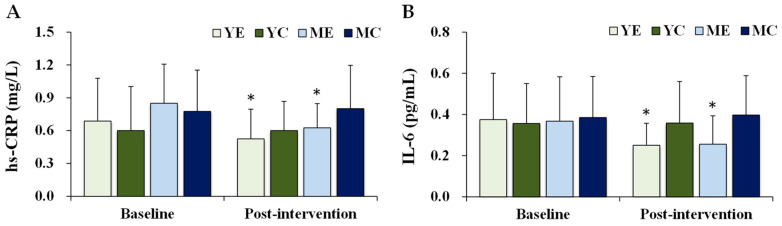
Changes in the plasma inflammatory biomarkers. Values are presented as mean ± standard deviation. (**A**) hs-CRP, high-sensitivity C-reactive protein; (**B**) IL-6, interleukin-6; YE, young exercise; YC, young control; ME, middle-aged exercise; MC, middle-aged control; * *p* < 0.05 vs. baseline within the same group.

Figure 4 shows the changes in the plasma inflammatory biomarkers. Significant time × group interactions were observed for hs-CRP (F (3, 28) = 3.942, *p* = 0.018, partial η^2^ = 0.297) and IL-6 (F (3, 28) = 3.744, *p* = 0.022, partial η^2^ = 0.253). Post hoc analyses indicated that both hs-CRP and IL-6 significantly decreased in the YE and ME groups compared to baseline (*p* < 0.05).

### 3.5. Changes in Intestinal Barrier Integrity Biomarkers

**Figure 5:**
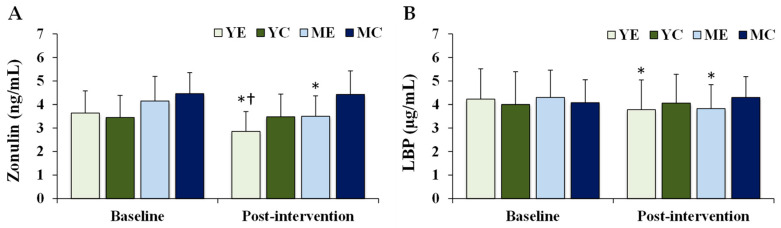
Changes in the plasma intestinal barrier integrity biomarkers. Values are presented as mean ± standard deviation. (**A**) zonulin; (**B**) LBP, lipopolysaccharide-binding protein; YE, young exercise; YC, young control; ME, middle-aged exercise; MC, middle-aged control; * *p* < 0.05 vs. baseline within the same group; ^†^ *p* < 0.05 vs. MC group.

Figure 5 shows the changes in the plasma intestinal barrier integrity biomarkers. Significant time × group interactions were observed for zonulin (F (3, 28) = 9.222, *p* < 0.001, partial η^2^ = 0.497) and LBP (F (3, 28) = 7.630, *p* = 0.001, partial η^2^ = 0.450). Post hoc analyses demonstrated that both zonulin and LBP significantly decreased in the YE and ME groups compared to baseline (*p* < 0.05). Post-intervention, zonulin levels in the YE group were significantly lower than those in the MC group (*p* < 0.05).

### 3.6. Correlations Among Baseline Biomarkers

Correlation analyses among baseline biomarkers revealed several significant associations (Table 2). Irisin showed strong positive correlations with TC (*r* = 0.74, *p* < 0.001), LDL-C (*r* = 0.65, *p* < 0.001), and HDL-C (*r* = 0.59, *p* < 0.001), as well as a moderate correlation with TG (*r* = 0.43, *p* = 0.005). Leptin was positively correlated with LBP (*r* = 0.54, *p* = 0.001) and IL-6 (*r* = 0.33, *p* = 0.048). Zonulin demonstrated positive correlations with TC (*r* = 0.59, *p* < 0.001), LDL-C (*r* = 0.61, *p* < 0.001), and irisin (*r* = 0.51, *p* = 0.002). Additionally, hs-CRP correlated with IL-6 (*r* = 0.39, *p* = 0.027). Collectively, these associations highlight the interconnected nature of organokines, lipid metabolism, systemic inflammation, and intestinal barrier function.

## 4. Discussion

It is widely recognized that obesity negatively affects body composition, inflammation, and intestinal barrier functioning [1,9]. However, evidence regarding the effects of exercise interventions on the intestinal barrier integrity, obesity-related inflammation, and metabolic abnormalities remains inconsistent [25,26]. Such variability may be influenced by age and individual characteristics, particularly in women undergoing pre- and post-menopausal transitions, during which hormonal changes alter body composition, systemic inflammation, mitochondrial function, and intestinal permeability [27,28,29]. Therefore, it is important to investigate the mechanism by which exercise modulates the expression of inflammatory organokines and intestinal barrier biomarkers in different age groups. Given that physiological responses to exercise vary with age [30,31,32] and that these differences are accentuated in women due to menopausal status and body composition, age-related effects warrant careful consideration. Therefore, this study examined the effects of an 8-week aerobic exercise program on body composition, lipid profiles, organokines (leptin and irisin), inflammatory markers (hs-CRP and IL-6), and intestinal barrier biomarkers (zonulin and LBP) in overweight and obese women stratified by age.

Consistent with previous findings that exercise improves body composition and VO_2_max regardless of obesity status [38], this study demonstrated significant reductions in body weight, BMI, BFM, and PBF, along with increases in LBM and VO_2_max in the YE and ME groups. These outcomes support earlier reports that moderate-intensity aerobic training (65% THR) effectively enhances body composition and cardiorespiratory fitness [39]. Improved VO_2_max is particularly relevant for obesity management because it contributes to higher daily energy expenditure and greater exercise efficiency [40]. Although baseline VO_2_max was higher in the younger groups (YE, YC), post-intervention improvements in both the YE and ME groups underscored the capacity of aerobic training to mitigate age-related declines in mitochondrial capacity, exercise performance, and metabolic efficiency [30]. Thus, aerobic training remains a potent intervention even in middle-aged obese women. In this study, VO_2_max was estimated using the Bruce Protocol, a widely applied treadmill test. While this method is validated, it provides only an estimation of maximal aerobic capacity. By contrast, cardiopulmonary exercise testing (CPET) with respiratory gas analysis is considered the gold standard, as it directly measures VO_2_max and provides additional insights into ventilatory thresholds, oxygen pulse, and metabolic flexibility, which are highly relevant to obesity and systemic inflammation [41,42]. Future studies should incorporate CPET to more comprehensively evaluate exercise capacity and cardiorespiratory health.

Lipid profile alterations are a hallmark of obesity and are typically characterized by elevated total cholesterol (TC), low-density lipoprotein cholesterol (LDL-C), and triglyceride (TG) levels, along with reduced high-density lipoprotein cholesterol (HDL-C) levels [43]. Aerobic exercise is known to decrease TC, LDL-C, and TG levels while increasing HDL-C levels [44,45,46]. In the present study, post-intervention TC, TG, and LDL-C levels were significantly reduced in the YE and ME groups, and TG levels were lower in the YE, YC, and ME groups than in the MC group. These results are consistent with previous evidence that aerobic training enhances lipid metabolism [44,45]. However, HDL-C levels did not change significantly, which may be attributable to the relatively short intervention duration (8 weeks). Consistent increases in HDL-C have generally been observed after at least 12 weeks of moderate-to-vigorous training [44], and meta-analyses have reported that aerobic exercise can significantly increase HDL-C by approximately 2–5 mg/dL [44]. In contrast, shorter interventions, such as 8 weeks, often fail to detect significant changes in HDL-C [45], suggesting that the duration applied in the present study may not have been sufficient to elicit measurable improvements. Moreover, HDL-C responsiveness is influenced by several factors, including baseline lipid status, exercise intensity, and dietary habits. Indeed, trials comparing moderate- and high-intensity exercise have reported more pronounced lipid improvements with higher-intensity protocols [47]. Taken together, the absence of significant changes in HDL-C observed in this study should be interpreted not as a lack of exercise effect, but rather as a result of limitations in intervention duration and intensity.

Organokines, including myokines, adipokines, and hepatokines, are bioactive proteins secreted by the skeletal muscles, adipose tissue, and liver [5,10,11]. Obesity is associated with increased pro-inflammation and decreased levels of anti-inflammatory organokines [10]. In this study, leptin significantly decreased in the YE and ME groups, consistent with a meta-analysis by Fontana et al. [48] showing that ≥180 min of moderate-intensity exercise per week for ≥8 weeks reduces leptin across weight categories. Irisin has been identified as a myokine that can promote browning of white adipose tissue, thereby enhancing energy expenditure and contributing to improvements in overweight and obesity [49]. However, in the present study, eight weeks of moderate-intensity aerobic exercise did not induce significant changes in circulating irisin. This result may be attributable to the combined influence of participant characteristics, such as sex, age, and degree of obesity, together with the exercise protocol applied. For example, a meta-analysis by Torabi et al. [49] reported that high-intensity interval training (HIIT) elicited greater increases in circulating irisin compared with aerobic training, with significant effects observed in overweight but not obese individuals, and in men but not women. In addition, accumulating evidence suggests that irisin tends to increase more robustly in response to resistance training than aerobic training, likely due to the greater muscular loading and metabolic stress imposed by resistance exercise [50,51,52]. Collectively, these findings imply that the absence of significant changes in irisin observed in this study may be related to the use of an eight-week moderate-intensity aerobic protocol, and that resistance or combined training modalities may be more effective in eliciting measurable irisin responses.

Systemic inflammation is a key feature of obesity-related complications [1,8,53]. Excessive adiposity promotes the secretion of TNF-α and IL-6, while reducing adiponectin, thus perpetuating a pro-inflammatory state [54]. In this study, hs-CRP and IL-6 levels significantly decreased in the YE and ME groups, in parallel with reductions in leptin and body fat. These findings suggest that aerobic exercise mitigates white adipose tissue-related inflammation, which is consistent with reports of positive correlations between hs-CRP, leptin, BMI, and body fat percentage [55]. Clinically, the reductions in hs-CRP (−0.16 to −0.23 mg/L) observed in the exercise groups are meaningful, as average values shifted further within the low-risk category defined by the AHA/CDC (<1, 1–3, >3 mg/L) [56]. Although participants were already at relatively low risk, further attenuation of systemic inflammation may translate into additional cardiometabolic benefits. IL-6 also decreased by approximately 0.11 pg/mL in both exercise groups. While no universally accepted cutoff exists for IL-6, cohort studies have demonstrated that even modest elevations predict increased risk of cardiovascular and metabolic disease [57,58], suggesting that the reductions observed here may carry clinical significance.

The intestinal barrier plays a crucial role in maintaining immune homeostasis. The disruption of epithelial integrity increases permeability, allowing bacterial products to translocate and promote endotoxemia [59]. Zonulin and LBP are representative biomarkers of barrier dysfunction [6,21]. Elevated zonulin facilitates the passage of luminal antigens into the circulation, contributing to systemic inflammation and metabolic disorders [60], while LBP enhances LPS–CD14–TLR4 interactions, initiating pro-inflammatory signaling [23]. In this study, both zonulin and LBP levels were significantly decreased in the YE and ME groups, and zonulin levels were lower in the YE group than in the MC group. These findings are consistent with those of Motiani et al. [20], Reljic et al. [61], and Bianco et al. [60], who reported that aerobic exercise reduces endotoxemia and improves intestinal integrity. Importantly, zonulin is not only a gastrointestinal marker but is also produced in extra-intestinal tissues, with elevated levels linked to obesity, metabolic syndrome, and systemic inflammation [60]. Thus, the reductions in body weight and improvements in VO_2_max observed here may have contributed to enhanced intestinal barrier function. Although standardized clinical thresholds for zonulin and LBP are lacking, the magnitude of reduction observed in this study (Zonulin: −0.65 to −0.79 ng/mL; LBP: –0.45 to –0.48 µg/mL) is comparable to differences reported between metabolically healthy and unhealthy obese populations in previous research. This suggests that aerobic exercise may exert clinically relevant effects on intestinal barrier function, further supporting its role as a non-pharmacological intervention for reducing obesity-related health risks.

Furthermore, correlation analyses among key biomarkers provided additional insight into their interrelationships. At baseline, irisin was positively correlated with TC, LDL-C, and HDL-C, supporting a potential link between myokine activity and lipid metabolism. Leptin showed positive correlations with LBP, hs-CRP, and IL-6, consistent with its role as an adipose-derived mediator of systemic inflammation. Zonulin was correlated with lipid parameters and irisin, suggesting a potential interplay between intestinal barrier function and metabolic regulation. Taken together, these associations reinforce the interpretation that aerobic exercise may influence interconnected pathways involving organokines, systemic inflammation, and intestinal permeability. Moreover, beyond statistical significance, the observed partial η^2^ values indicated large effect sizes for most outcomes, suggesting that the intervention exerted substantial practical impact. These findings are consistent with previous aerobic exercise interventions in overweight and obese populations, which have reported large effect sizes for improvements in body composition, cardiorespiratory fitness, and inflammatory markers [36,62].

Taken together, the present findings demonstrate that aerobic training improves body composition, lipid metabolism, systemic inflammation, and intestinal barrier integrity in young and middle-aged obese women. These results underscore the potential of structured aerobic exercise as a practical strategy for mitigating obesity-related risks and promoting metabolic health across different age groups, including women undergoing menopausal transition. This study provides novel evidence regarding the effects of aerobic exercise on organokines, systemic inflammation, and intestinal barrier function in overweight and obese women. Unlike previous studies that mainly focused on either metabolic or inflammatory outcomes, our findings demonstrated that aerobic exercise simultaneously improved body composition, lipid metabolism, and organokine regulation, while reducing intestinal permeability. Importantly, this study directly compared young and middle-aged women, showing that the beneficial effects of aerobic exercise were consistent across age groups, thereby contributing new insights into the generalizability of exercise-induced health benefits. A notable strength of this study is that participants were stratified not only by age but also by menopausal status, with the young group consisting of premenopausal women and the middle-aged group consisting of postmenopausal women. This classification increases the relevance of the findings by accounting for physiological differences associated with menopausal transition.

Several limitations should be acknowledged. First, although participants were instructed to maintain their habitual diet and to refrain from alcohol consumption during the intervention, detailed dietary intake was not recorded. Future studies should include more rigorous dietary control or dietary monitoring, as diet strongly affects lipid profiles, inflammatory markers, and gut permeability. Second, participants were advised to avoid any additional structured exercise outside the intervention; however, incidental daily physical activity was not objectively monitored (e.g., with accelerometers). Future research should consider objectively monitoring daily activity levels to better account for variations in physical activity outside the intervention. Third, although the sample size (*n* = 32) was relatively small, statistical power analysis confirmed adequacy for detecting both main and interaction effects; nonetheless, larger studies are warranted to enhance the generalizability of the findings. Fourth, the 8-week intervention period and the absence of follow-up assessments limited the ability to determine the long-term sustainability of exercise-induced adaptations. Future studies should employ longer intervention periods (e.g., >12 weeks) and include follow-up evaluations to better assess the persistence of metabolic and physiological benefits. Fifth, body composition was measured using bioelectrical impedance analysis, which is a practical and widely used method in field-based research. However, future studies may benefit from using more precise techniques, such as dual-energy X-ray absorptiometry (DEXA), to provide a more detailed assessment of body composition. Sixth, waist circumference was not measured, limiting the assessment of central adiposity—an important indicator of cardiometabolic risk. Future studies should include this parameter to better evaluate abdominal fat distribution. Seventh, VO_2_max was estimated using the Bruce Protocol rather than cardiopulmonary exercise testing (CPET); however, the procedure was standardized and closely supervised, minimizing potential measurement bias. Eighth, only leptin and irisin were analyzed as representative organokines, providing a limited view of the broader network of adipokines, myokines, and hepatokines. Future research should expand the biomarker panel to offer deeper mechanistic insights into exercise-induced metabolic regulation. Finally, as the study included only overweight and obese women, the findings may not be directly generalizable to men or mixed populations.

## 5. Conclusions

This study demonstrated that aerobic exercise improves body composition, cardiorespiratory fitness, lipid profiles, and leptin levels in overweight and obese women, while reducing systemic inflammation. Aerobic exercise was also associated with favorable changes in circulating biomarkers of intestinal barrier function. The observed partial η^2^ values indicated large effect sizes, underscoring the practical impact of the intervention. These findings highlight aerobic training as a promising non-pharmacological strategy for obesity management. However, given the small sample size, short intervention duration, and absence of dietary and daily activity monitoring, the results should be interpreted with caution, particularly regarding subtle age-related differences. While strengths include the randomized controlled design and comprehensive biomarker assessment, these limitations must be considered. Future research should clarify the underlying mechanisms and evaluate sex- and age-specific differences in exercise responses with larger and longer-term studies.

## Figures and Tables

**Table 1:** Baseline physical characteristics of participants.

Variables	YE (*n* = 8)	YC (*n* = 8)	ME (*n* = 8)	MC (*n* = 8)	Value ^#^
Age (years)	23.50 ± 3.51	25.00 ± 4.96	56.63 ± 4.31	56.75 ± 2.92	F = 175.799 *p* < 0.001
Height (cm)	161.25 ± 3.98	159.41 ± 4.42	159.31 ± 3.46	156.10 ± 4.56	F = 2.151 *p* = 0.116
Weight (kg)	65.25 ± 9.73	67.12 ± 8.07	66.06 ± 6.16	61.71 ± 3.97	F = 0.827 *p* = 0.490
BMI (kg/m^2^)	25.13 ± 4.03	26.40 ± 2.69	26.31 ± 1.84	25.35 ± 1.60	F = 0.363 *p* = 0.780
BFM (kg)	24.62 ± 6.18	26.00 ± 6.13	24.30 ± 3.55	23.86 ± 3.11	F = 0.278 *p* = 0.841
LBM (kg)	21.97 ± 2.75	22.68 ± 2.24	22.65 ± 2.55	20.33 ± 1.23	F = 1.881 *p* = 0.156
HRmax (bpm)	186.63 ± 6.44	182.00 ± 11.17	164.25 ± 11.22	163.69 ± 10.92	F = 10.951 *p* < 0.001

Values are presented as mean ± standard deviation. YE, young exercise; YC, young control; ME, middle-aged exercise; MC, middle-aged control; BMI, body mass index; BFM, body fat mass; LBM, lean body mass; HRmax, maximum heart rate; ^#^ value determined using one-way ANOVA for between-group comparisons at baseline.

**Table 2:** Pearson correlation coefficients among baseline biomarkers.

Variables	Irisin	Leptin	TC	TG	LDL-C	HDL-C	Zonulin	LBP	hs-CRP	IL-6
Irisin	1.00	0.48	0.74	0.43	0.65	0.59	0.51	0.50	0.23	0.27
Leptin		1.00	0.54	0.36	0.38	0.45	0.44	0.54	0.19	0.33
TC			1.00	0.57	0.87	0.72	0.59	0.55	0.26	−0.02
TG				1.00	0.59	0.28	0.59	0.30	0.12	0.1
LDL-C					1.00	0.47	0.61	0.41	0.13	0.03
HDL-C						1.00	0.37	0.44	0.18	0.29
Zonulin							1.00	0.48	0.21	0.15
LBP								1.00	0.24	0.27
hs-CRP									1.00	0.39
IL-6										1.00

## Data Availability

The original contributions presented in this study are included in the article. Further inquiries can be directed to the corresponding author.
